# Towards Simazine Monitoring in Agro-Zootechnical Productions: A Yeast Cell Bioprobe for Real Samples Screening

**DOI:** 10.3390/bios8040112

**Published:** 2018-11-15

**Authors:** Gerardo Grasso, Ludovico Caracciolo, Giulia Cocco, Chiara Frazzoli, Roberto Dragone

**Affiliations:** 1 Istituto per lo Studio dei Materiali Nanostrutturati, Consiglio Nazionale delle Ricerche, P.le Aldo Moro 7, 00185 Roma, Italy; ludovico.caracciolo@hotmail.it (L.C.); dr.giulia.cocco@gmail.com (G.C.); roberto.dragone@ismn.cnr.it (R.D.); 2 Dipartimento di Scienze e Tecnologie per l’Agricoltura, le Foreste, la Natura e l’Energia, Università degli Studi della Tuscia, 01100 Viterbo, Italy; 3 Dipartimento Malattie Cardiovascolari, Dismetaboliche e dell’Invecchiamento, Istituto Superiore di Sanità, Via Giano della Bella 34, 00162 Roma, Italy; chiara.frazzoli@iss.it

**Keywords:** biosensoristic devices, simazine, milk chain, livestock drinking water, precision livestock farming, One Health

## Abstract

Simazine is an herbicide that is able to contaminate surface waters, ground waters, and milk/dairy products, thus posing concerns in both environmental health and food safety. A yeast-based bioprobe was utilized to detect simazine in spiked real samples of livestock drinking water and raw cow’s milk. Yeast aerobic respiration was taken as short-term toxicological endpoint. We carried out comparative measures of yeast oxygen consumption between simazine-spiked samples and blank samples. Percentage interference (%ρ) on yeast aerobic respiration was calculated through the comparison of aerobic respiration of simazine-exposed and non-exposed yeast cells. The method was optimized for raw cow’s milk samples by using boric acid as fungistatic agent in order to avoid cellular proliferation. Overall, the results have shown that simazine can be detected up to concentrations five times below the EU legal concentration limits for drinking water (0.02 ppb) and cow’s milk (2 ppb) (%ρ values of 18.53% and 20.43% respectively; %RSD ≤ 15%). Dose-effect relationships of simazine were assessed. The findings of the bioassays match reasonably well with known mechanisms of toxicity and intracellular detoxification in yeast. A correlation between fat content in milk samples and analytical performance of the bioprobe was established. Results suggest the involvement of a matrix effect, presumably due to lipid sequestration of simazine. The yeast-based bioprobe has proved to be sensitive and suitable for the detection of simazine in real samples in concentrations of interest.

## 1. Introduction

The chloro-s-triazines is an important family of pre-emergence herbicides extensively used for weed control in crop areas of e.g., corn, sorghum, sugarcane, orchards, and perennial crops, well as in non-crop areas. In particular, simazine (6-chloro-*N*,*N*′-diethyl-1,3,5-triazine-2,4-diylamine) is one of the most relevant members of chloro-s-triazines, as it is an herbicide additionally used as a desiccant, defoliant and as algaecide for the aquatic weed control [1]. Simazine is a chemical of great agronomical importance worldwide. In fact, even though simazine use as herbicide was banned in the European Union [2], it is still utilized in many countries, including the United States (in particular in California) [3], Canada, Brazil, and China (the world’s largest producer and consumer of simazine) [4]. Simazine is moderately persistent in the environment [5], and due to its water solubility (6.2 mg/L) and its relatively low soil adsorption coefficient, (*K*_oc_ = 130), it is likely to contaminate both surface and groundwater. Indeed, simazine is the second most commonly detected pesticide in surface and ground waters in the United States, Europe, and Australia [1]. Since 2005, simazine is no more in production in Italy, but it is still present in Italian natural waters [6]. 

Simazine can carry over in foods of animal origin, e.g., in milk from cows consuming contaminated forage crops and/or drinking contaminated water [7,8,9]. Environmental and food contamination by simazine raises concern over public health. Indeed, together with toxicity associated with the inhibition of photosynthesis and grana thylakoid damage [10], other toxic effects on non-target organisms are reported in literature, including endocrine-disrupting effects [11,12,13], in vitro neurotoxicity [14], oxidative stress in common carp [15], and toxicosis in sheep and cattle [16,17]. 

Against this background, the availability of reliable screening methods for the on-site detection of simazine residues in environmental and food matrices is of utmost importance. In recent literature, different screening methods for simazine detection in water samples based on chromatographic methods coupled to time-of-flight mass spectrometry have been described [18,19,20,21,22]. Although these methods are highly sensitive, they often require sample pretreatment steps, are costly, and require bulky equipment; furthermore, only highly skilled personnel can perform the analyses. A flow injection chemoluminescence-based method has also been developed for triazines detection in natural waters [23]. However, a solid phase extraction step was required to remove the interference of natural organic matter and concentrate the samples. 

During the last few years, several sensor-based detecting methods and devices have been developed for the detecting of pesticide residues or biocidal active substances (including insecticides, fungicides, and herbicides) [24,25,26]. Biosensoristic devices (i.e., sensoristic devices that use biological responsive elements) are promising tools with interesting analytical features, which can be potentially exploited for on-site field applications for both environmental and food matrices (e.g., agricultural waters and cow milk) [27]. Whole cell-based biosensoristic devices are characterized by a different analytical approach compared to other biosensoristic devices and chemical analyses [28]. Through the selection of a given cellular function and an appropriate short-term in vitro biochemical endpoint, the use of intact biological entities allows a rapid assessment of levels of different chemical contaminants (in structure and in type) based on bioactivity on the selected cellular function. This approach is more useful for environmental/food safety risk assessments than a ‘substance-by-substance’ approach that may result in underestimations of risks and real levels of chemical contamination. A microalgae whole cell-based, optic biosensoristic device has been previously tested for simazine detection, but not in real environmental or food samples [29].

The budding yeast *Saccharomyces cerevisiae* is a well-consolidated eukaryotic model organism for genetic and toxicology studies [30]. Cellular aerobic respiration has been shown to be a susceptible target of bioactivity of several organic and inorganic chemical contaminants (e.g., herbicides, surfactants and heavy metals). In our previous works, interference on cellular aerobic respiration in *S. cerevisiae* were utilized as the integral toxicity index for the detection of platinum-group elements [31], wood preservatives [32], and herbicide diuron [33]. 

In this work, a yeast-based biosensoristic device (hereinafter referred to as ‘bioprobe’) was used in respirometric bioassays to detect simazine in real samples of livestock drinking water and raw cow’s milk. 

## 2. Materials and Methods

### 2.1. Simazine Solution

Simazine solutions were verified through spectrophotometric measurements (UV2 UV/Visible, Unicam Instruments, Cambridge, UK) at the fixed wavelength of λ = 224 nm, i.e., the wavelength of maximum absorbance for simazine [34]. Baseline measurement were performed at λ = 224 nm on methanolic aqueous solution. The latter was also used as reference for simazine solution measurements. All measurements were carried out in quartz cuvettes with 1 cm optical path lengths. All solutions were kept in the dark and stored at 4 °C. The simazine concentrations tested are indicated in Table 1.

### 2.2. Other Chemical Solutions

For bioassays in water samples, a 3 mol/L stock solution of glucose was prepared in high-purity deionized water (Milli-Q system, Merck Millipore, Billerica, MA, USA) from glucose D (+) 99.5% GC (Sigma Aldrich, St. Louis, MO, USA). Aliquots of glucose stock solution were added to each tested water sample as the carbon source for *S. cerevisiae* cells (final concentration 0.5 mol/L glucose).

For bioassays in raw cow’s milk, no glucose solution addition was required. A 3.2% *w*/*v* boric acid stock solution (BioReagent, for molecular biology, suitable for cell culture, suitable for plant cell culture, ≥99.5%, Sigma Aldrich) was prepared in high-purity deionized water. Aliquots of stock solution (0.480 mL) were added to each tested raw cow’s milk samples (final concentration of 0.16%) to prevent *S. cerevisiae* proliferation during the measurements.

### 2.3. Samples

Real samples were collected from ‘Elio Pascolini’ dairy farm (Lazio region, 41°54′47.94″ N, 12°15′48′25″ E). Livestock drinking water was manually sampled from water troughs by placing under the tap sterile Pyrex glass bottles according to the ISO standard (ISO/TC 147/SC 6/WG1 Sampling management and ISO/TC 147/SC 6/WG 3 Preservation and handling of samples). Raw cow’s milk was manually sampled from the refrigerated milk-farm bulk tank. To ensure sample homogeneity, milk was agitated for at least 10 min before sampling, and it was collected in sterile Pyrex glass bottles [35]. Before each bioassay, samples were kept cooled (4 °C), and they were tested within 24 h. Chemical analysis were performed to test for the presence of heavy metals, pesticides, and mycotoxins. All the substances were below the legal thresholds (Tables S1 and S3). For comparative tests, commercial UHT milk (whole, semi-skimmed and skimmed) was purchased in a local supermarket (chemical composition is reported in Table S2).

### 2.4. S. cerevisiae Suspensions

Yeast suspensions (500 mg/mL) were prepared daily starting from 50 mg ± 1.0 mg dried yeast (*Saccharomyces cerevisiae* yeast, Type II, Sigma Aldrich) rehydrated with 10 mL of Milli-Q water in sterilized test tubes 12 h before the bioassay. The most appropriate cellular concentration of yeast stock suspension used in the present work (1.5 × 10^8^ colony forming unit/mL or cfu/mL) and the 1:100 dilution ratio for the bioassays were defined by preliminary tests at different *S. cerevisiae* cells/simazine dose ratios. Indeed, sensitivity and limit of detection (LoD) of a cytotoxicity assay mainly depend from the number of cells/chemical contaminant dose ratio [36]. Turbidimetric measurements of optical density at the wavelength of 525 nm (OD_525nm_) were performed on eight serial dilutions of a yeast cell stock suspension (yeast stock suspension 1.5 × 10^8^ cfu/mL) in quartz cuvettes with 1 cm optical path lengths against water as reference (UV2 UV/Visible, Unicam Instruments, Cambridge, UK). Before turbidmetric measurement, for each dilution cell, counting was carried out using a counting chamber and a Reichert-Jung MicroStar 110 microscope, Leica Microsystems Wetzlar, Germany (range of colony-forming unit/mL between 5 × 10^5^–5 × 10^6^ cfu/mL). The calibration curve was constructed by plotting OD_525nm_ vs. cfu/mL values (y = 10^−7^ × −0.0001), and good agreement (R^2^ = 0.9986) was found between OD_525nm_ and yeast cellular concentration (Figure S1).

### 2.5. Yeast Cell Proliferation Control

Non-proliferation condition represents a key aspect of the respirometric bioassay: indeed, the variation of dissolved O_2_ between the two steady states is linked to cellular O_2_ consumption rate of yeast suspension, and it is proportional to cell concentration. Thus, if the cell population remains constant during the bioassay running time, any variation of this parameter for the exposed cellular suspensions compared with the control (in the same non-proliferating condition) can be associated with an interference with cellular aerobic respiration. For livestock drinking water samples, no significant cellular proliferation during bioassays has occurred *(*ΔOD_525nm_ ≤ 0.05 a.u.). Conversely, cow’s milk can support *S. cerevisiae* yeast growth [37], as we also confirmed by a set of preliminary bioassays. In order to guarantee the non-proliferation condition for the 2 h. running time of the test, the effectiveness of boric acid (H_3_BO_3_) as fungistatic agent [38] was tested for milk samples. Three final concentrations of boric acid (0.10, 0.16% and 0.20% *w*/*v*) were tested on raw milk samples adding aliquots of a 3.2% *w*/*v* boric acid stock solution and 0.150 mL of 500 mg/mL *S. cerevisiae* cell suspension (1.5 × 10^8^ cfu/mL). O_2_ consumption was monitored for 12 h and it was compared with O_2_ consumption in control raw milk samples (without boric acid).

### 2.6. Bioassays

A yeast-based probe involves two main components: (i) *S. cerevisiae* cell suspension as biological recognition element (0.150 mL of 500 mg/mL yeast cell suspension added to each samples), (ii) Clark-type oximeter (model 360, Amel Instruments S.r.l., Milano, Italy) as amperometric transducer, to measure instantly changes in dissolved oxygen (O_2_) concentration in tested samples. Two-point calibration of Clark-type oximeters was done before every test, as described in Dragone et al. 2015 [33]. 

The array of yeast-based bioprobe (Figure S2) was assembled in an open system (i.e., oxygen into the samples was in equilibrium with the oxygen in the air). Samples were placed into open measurement glass chambers (25 mL), and thermostat controlled (28.0 ± 0.1 °C) under constant magnet stirring (200 rpm).

The experimental set-up of each bioassay included two groups of samples.

Livestock drinking water samples

- Blank samples: 12.500 mL sample + 2.500 mL of 3 mol/L glucose solution + 0.150 mL of methanolic solution (concentration < 0.1%) without simazine
- Simazine-spiked samples: 12.500 mL sample + 2.500 mL of 3 mol/L glucose solution + 0.150 mL methanolic solution (concentration < 0.1%) of simazine

Raw cow’s milk samples

- Blank samples: 13.875 mL of sample + 0.750 mL of 3.2% *w/v* boric acid solution + 0.375 mL of methanolic solution (concentration < 0.1%) without simazine
- Simazine-spiked samples: 13.875 mL of sample + 0.750 mL of 3.2% *w/v* boric acid solution + 0.375 mL methanolic solution (concentration < 0.1%) of simazine

**Figure 1:**
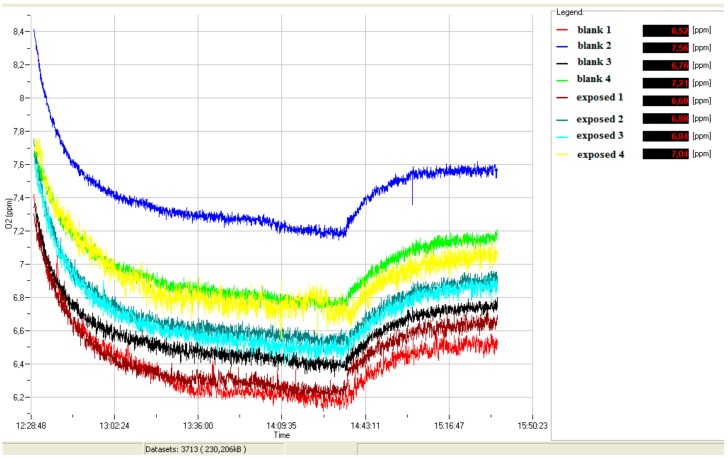
Exemplary curve of dissolved oxygen (ppm O_2_) vs. time.

During the bioassay, an experimental respirometric curve (dissolved O_2_ vs. time) for blank and simazine spiked samples (see example in Figure 1) was acquired through a continuous data logger.

**Figure 2:**
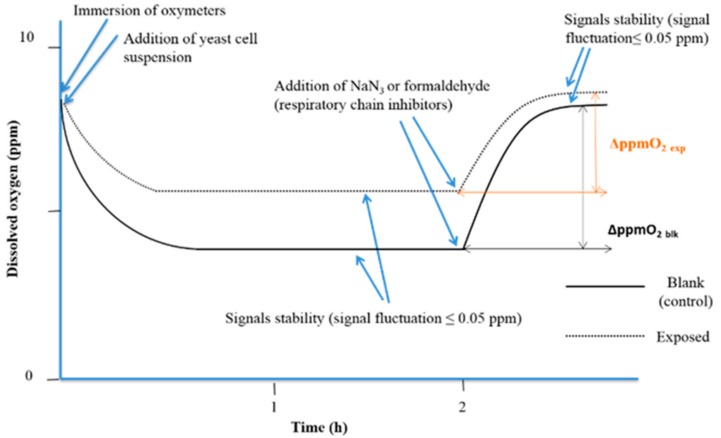
The experimental respirometric curve shows the dissolved oxygen (ppm O_2_) vs. time (adapted from Dragone et al., 2015 [33]).

When the cellular oxidation rate equalizes the O_2_ diffusion rate from the air, a stationary state is reached (signal stability with fluctuation less than 0.05 ppm). After 2 h. of exposure to simazine, 150 µL of 0.2 mol/L sodium azide (NaN_3_ ≥ 99.0%, AMS Biotechnology Ltd., Bioggio, Switzerland) was added to fully inhibit the yeast cell respiration. In water samples, same aliquot of formaldehyde (ACS reagent, 37 wt. % in H_2_O, Sigma Aldrich) was added. The interruption of cellular O_2_ consumption triggers a progressive increase in dissolved O_2_, up to a second steady state that corresponds to the amount of dissolved O_2_ in absence of yeast cellular O_2_ consumption. The analytical parameter used was the variation of the dissolved O_2_ (ΔppmO_2_) between the two steady states of the experimental curves before and after the addition of respiratory chain inhibitor (NaN_3_ or formaldehyde) (Figure 2). 

Means of readings of the dissolved O_2_ concentration at 10 min before respiratory chain inhibitor addition (first plateau), and once the second plateau was reached, were calculated, and ΔppmO_2_ was calculated for each experiment. The percentage interference of cellular respiration (%ρ) was calculated with the following algorithm:

%ρ = [1 − (ΔppmO_2 exp_/ΔppmO_2 blk_)] × 100 (1)

where ΔppmO_2 exp_ = mean of variations of the dissolved O_2_ (in ppm) in fortified samples, and ΔppmO_2 blk_ = mean of variations of the dissolved O_2_ (in ppm) in blank samples. All experiments were repeated at least four times (each control and exposed samples had four replicates for each experiment). To assess the repeatability of the measurements, four runs of the array measurements were performed on the same day, whereas reproducibility was verified on three different days starting from freshly prepared solutions and yeast suspensions. Relative standards deviation percentages were calculated (%RSD calculated ≤ 15%). Data were statistically analyzed with ANOVA Randomized Block Design. 

#### Sample Matrix Effect: Influence of Milk Fat Content 

To evaluate the masking effect of the fat content of milk on simazine, comparative 2 h respirometric bioassays on commercial UHT milk (full fat, semi-skimmed and skimmed UHT pasteurized and microfiltered milk) were performed. In particular, UHT milk samples were fortified with simazine working solution to final concentration of 10 ppb (EU legal limit for simazine residue in cow’s milk) and tested with the yeast- based bioprobe.

UHT milk samples

- Blank samples: 13.875 mL of sample + 0.750 mL of 3.2% *w/v* boric acid solution + 0.375 mL of methanolic solution (concentration < 0.1%) without simazine
- Simazine-spiked samples: 13.875 mL of sample + 0.750 mL of 3.2% *w/v* boric acid solution + 0.375 mL methanolic solution (concentration < 0.1%) of simazine (final concentration 10 ppb)

## 3. Results

The results of comparative respirometric bioassays in raw cow’s milk samples in the presence of different selected concentration of boric acid (0.10, 0.16% and 0.32% *w*/*v*) allowed us to identify 0.16% *w*/*v* concentration as the minimum boric acid concentration that exerts a fungistatic action for 12 h. During the 12 h O_2_ consumption monitoring, signal fluctuations registered from milk samples containing 0.16% *w*/*v* boric acid were less than 0.10 ppm. Repeatability of the measurements was assessed performing four runs of the array measurements on the same day. Reproducibility was verified on three different days starting from freshly prepared solutions and yeast suspensions. Relative standards deviation percentages were calculated (%RSD calculated ≤ 15%). Data were statistically analyzed with ANOVA Randomized Block Design for statistical differences between means values of Δppm O_2 exp_ (milk samples with boric acid) and Δppm O_2 blk_ (milk samples without boric acid) (*p* < 0.05). Considering these findings, we have used the final concentration of 0.16% *w*/*v* boric acid for all the bioassays on raw cow’s milk.

**Figure 3:**
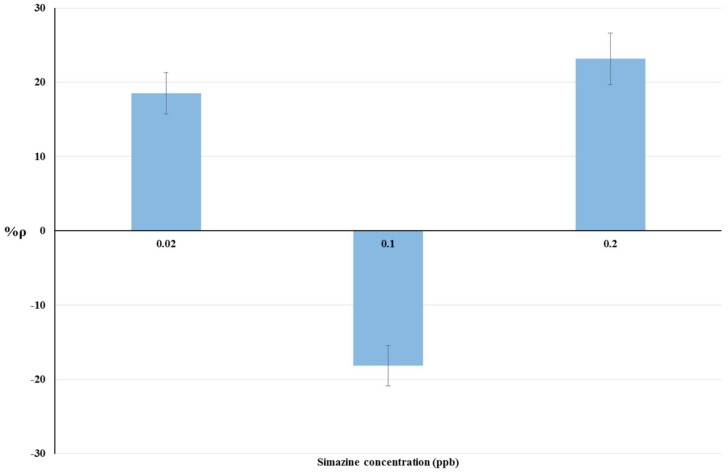
Dose-effect histograms for 2 h exposure to simazine in livestock drinking water samples. %RSD ≤ 15%; ANOVA testing using Randomized Block Design was applied for statistical differences between means values of Δppm O_2_ exp and Δppm O_2_ blk (*p* < 0.05).

**Figure 4:**
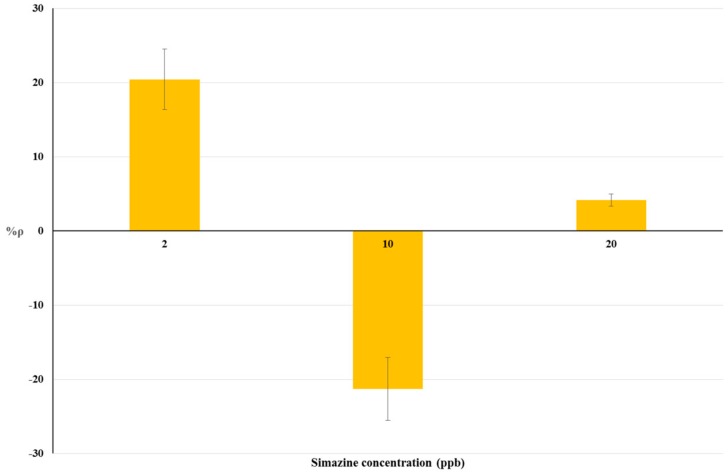
Dose-effect histograms for 2 h exposure to simazine in raw cow’s milk samples. %RSD ≤ 15%; ANOVA testing using Randomized Block Design was applied for statistical differences between means values of Δppm O_2 exp_ and Δppm O_2 blk_ (*p* < 0.05).

The results of bioassays on livestock drinking water samples and raw cow’s milk samples are summarized in Table 2. The relationship between simazine concentration and %ρ is shown by histograms in Figure 3 and Figure 4 respectively. Statistical tests were done using ANOVA testing for Randomized Block Design, and a significant relationship between fortified and blank samples was found for all concentrations tested (*p* < 0.05).

Non-linear trends of %ρ values were observed for both matrices tested (Figure 2 and Figure 3). Positive %ρ values indicate a decrease-related inhibition of cellular respiration, while negative %ρ values indicate an increase in cellular O_2_ consumption due to hyperstimulation. These aspects are detailed in the Discussion section. 

**Figure 5:**
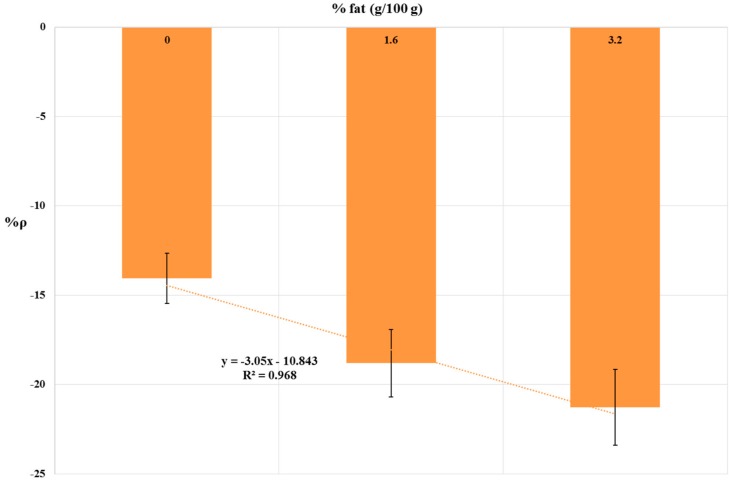
Dose-effect histogram for 2 h exposure to 10 ppb simazine in commercial whole, semi-skimmed and skimmed UHT milk. %RSD ≤ 15%; ANOVA testing using Randomized Block Design was applied for statistical differences between means values of Δppm O_2 exp_ and Δppm O_2 blk_ (*p* < 0.05).

The results of 10 ppb simazine detection in UHT milk samples are summarized in Table 3. The relationship between fat content and %ρ values is shown by histograms in Figure 5. A linear correlation (R^2^ = 0.968) between fat content and %ρ values were found. Statistical tests were done using ANOVA testing for Randomized Block Design, and a significant relationship between exposed and control samples was found for all UHT milk samples tested (*p* < 0.05). 

## 4. Discussion

### 4.1. Yeast Cell Proliferation Control

As previously described by Schmidt et al. 2010 [38], 0.31% *w*/*v* boric acid was the minimal concentration to exert a fungistatic action on *S. cerevisiae* for three-days growth in yeast extract peptone dextrose (YPD) medium at 30 °C. Our findings suggest that 0.16% *w*/*v* boric acid can exert a fungistatic action on *S. cerevisiae* for 12 h. incubation in raw cow’s milk at 28 °C. The discrepancy between the results may be due to differences in experimental conditions. Indeed, Schmidt et al. 2010 [38] performed a three days-long test in YPD medium, a complete medium optimal for yeast growth. Thus, in YPD medium, a higher concentration of boric acid (0.31% *w*/*v*) could be required than in cow’s milk (0.16% *w*/*v*). 

Testing of other well-known fungistatic agents like benzoate, sorbate, and azole derivatives was not carried out because of the effects on metabolism and mitochondrial respiration of yeast, as previously described in the literature [39,40,41]. Indeed, these effects could negatively affect the performances of the bioassay, interfering with *S. cerevisiae* response to simazine. According to Smith et al., 2010, boric acid only interferes with cytokinesis [38]. We confirmed that, in our experimental conditions, no mitochondrial interferences occur during 12 h exposure of *S. cerevisiae* to 0.16% *w*/*v* boric acid. Overall, the use of boric acid as fungistatic agent is a suitable and cost-effective optimization of the method for bioassays in raw cow’s milk samples.

### 4.2. Bioassays on Livestock Drinking Water and Raw Cow’s Milk Samples

The dose-response histograms for bioassays on livestock drinking water and raw cow’s milk sample spiked with simazine (concentration from five-times below to two-fold above the EU legal limits) are showed in Figure 3 and Figure 4 respectively. Similar non-linear dose–response trends of %ρ values (changes from positive to negative values) with increased simazine concentrations can be observed for both water and milk samples.

According to our interpretation, %ρ values may originate from the combined action of three different mechanisms, i.e., inhibition of cellular respiration, hyperstimulation, and detoxification/adaptive responses. Its own dose-dependent priming and action characterize each of these mechanisms. Therefore, %ρ values should be viewed as a “snapshot” of yeast cellular respiration at 2 h exposure to a certain concentration of simazine. The combination of toxic mechanisms and adaptive response mechanisms in *S. cerevisiae* exposed to simazine may influence not only %ρ values, but also the changes from positive to negative %ρ values. Such combinations could result in a defined exposure-response pattern, as shown in Figure 3 and Figure 4.

In our previous works, we assumed similar combinations of toxic mechanisms and adaptive response mechanisms in *S. cerevisiae* exposed to platinum group elements [31] and herbicide diuron [33]. Our hypotheses were supported by literature data about the mechanisms involved. Concerning the present work, inhibition of cellular respiration, hyperstimulation of cellular respiration, and detoxification/adaptive responses have also been previously described in the literature for simazine exposure, as well as for *S. cerevisiae*.

Positive %ρ values indicate a lower O_2_ consumption by yeast cell in simazine-spiked samples (compared to blank samples). These results may be ascribed to the inhibition of cellular respiration by hyperproduction of reactive oxygen species (ROS), inhibition of cellular antioxidant capacity, oxidative damage to lipids and proteins, and interference of ATP synthesis [15,42,43]. Negative %ρ values indicate a higher O_2_ oxygen consumption by yeast cell in simazine-spiked samples (compared to blank samples). Concerning this effect, preliminary tests have proven that in our experimental conditions, yeast cells are in an optimal physiological state for cellular aerobic respiration, and therefore, yeast cells exhibit a maximum O_2_ consumption rate. Thus, our results may be ascribed to the uncoupling of oxidative phosphorylation by simazine. Uncouplers are chemicals that inhibit the coupling between the electron transport and phosphorylation, dissipate the electrochemical proton gradient across the inner mitochondrial membrane, and lead to increased oxygen consumption and inhibition of ATP synthesis [44]. Previous works have suggested that simazine may also acts as an uncoupler of oxidative phosphorylation [45,46], but the mechanism has not been characterized so far. Concerning detoxification/adaptive response to herbicides in yeast cells, in *S. cerevisiae*, at least two plasma-membrane transporters and multidrug extrusion pumps ATP-binding cassette or ABC transporter, pleiotropic drug resistance protein 5 (Pdr5p) and the Major Facilitator Superfamily multidrug resistance (MFS-MDR), Tpo1p are involved in resistance to simazine [47,48]. Considering the ATP dependence of ABC transporters to work as extrusion pumps, and that cited mechanisms of inhibition and hyperstimulation of cellular respiration lead to depletion of intracellular ATP, we can hypothesize a possible influence of cellular ATP content on the %ρ changes observed, but no data are available so far, and further measurements will be necessary.

#### Sample Matrix Effect: Influence of Milk Fat Content

The octanol/water partition coefficient (Kow) is used as an indicator of hydrophobicity of organic compounds, and thus, the partitioning of a chemical between aqueous and lipid phases [49]. Simazine is a moderately hydrophobic compound (log Kow of 2.18 at 25 °C), and the association of simazine with the lipid fraction of milk has been previously verified [9]. To verify the influence of milk fat content on analytical performance of the yeast-based bioprobe, a 10 ppb simazine concentration was chosen, because it is the EU maximum residue limit for simazine in cow’s milk. The results (shown in Figure 5) suggest a moderate masking effect of simazine by milk fat. Barchanska et al., 2012 [50] have obtained similar results with atrazine (a chloro-s-triazine related to simazine, with log Kow 2.61 at 25 °C) in milk samples using an enzyme—linked immunosorbent assay (ELISA). Indeed, when milk samples had been spiked with 10 ppb atrazine, the recovery was 97% with skimmed milk (0% fat) and 86% for whole milk (3.2% fat) [50]. In general, bovine milk typically contains 3–4% fat. Fat levels in milk may be influenced by several factors, including cow breed, stage of lactation, season, feed, and milking procedures; however, typically, fat percentage is not lower than 2% [51]. In our experimental conditions, %ρ values were not significantly different from semi-skimmed milk samples (1.6% fat) and whole milk samples (3.2% fat) (%ρ = −18.81%; %ρ = −21.28% respectively; %RSD ≤ 15%). Thus, influence of average milk fat content on the analytical performance of the yeast-based bioprobe could be considered to be negligible.

## 5. Conclusions

The potential use of the yeast-based bioprobe as a screening method for the detection of simazine in real samples was verified. In particular, the proposed bioprobe is able to detect simazine in real samples at concentrations up to five times below those of EU legal concentration limits, i.e., 0.02 ppb in drinking water and 2 ppb in cow’s milk. 

A first pattern of cellular response in the presence of simazine at concentrations of interest has been presented. In future work we may carry out a more complete assessment of cellular response patterns and bioprobe performances in real samples. 

Overall, these findings broaden the range of chemicals detectable by the yeast-based bioprobe, and confirm the suitability of this bioprobe to be employed as an integral toxicity monitoring system for environmental and food surveillance at critical control points for safeguarding animal welfare and food safety. 

Furthermore, in future studies, we plan to validate the system, and automate/integrate the yeast-based bioprobe in the technological platform BEST (PCT WO/2010/001432): (Bio)Sensors’ system in Food Safety.

## Figures and Tables

**Table 1:** Simazine concentrations tested (in µg/L or ppb). The EU legal limits for simazine residue in water and milk are indicated in bold.

Livestock Drinking Water	Raw Cow’s Milk
0.02	2
**0.1 ^1^**	**10 ^2^**
0.2	20

^1^ Council Directive 98/83/EC of 3 November 1998 on the Quality of Water Intended for Human Consumption. Available online: http://eur-lex.europa.eu/legal-content/EN/TXT/?qid=1405609372533&uri=CELEX:31998L0083. ^2^ Commission Regulation (EU) No 310/2011 of 28 March 2011 amending Annexes II and III to Regulation (EC) No 396/2005 of the European Parliament and of the Council as regards maximum residue levels for aldicarb, bromopropylate, chlorfenvinphos, endosulfan, EPTC, ethion, fenthion, fomesafen, methabenzthiazuron, methidathion, simazine, tetradifon and triforine in or on certain products. Available online: http://eur-lex.europa.eu/LexUriServ/LexUriServ.do?uri=OJ:L:2011:086:0001:0050:EN:PDF.

**Table 2:** Simazine concentrations tested (in ppb) in livestock drinking water and raw cow’s milk samples with the corresponding means values of indexes of respiratory inhibition (%ρ). The EU legal limit for simazine residue in each matrix is indicated in bold.

Livestock Drinking Water		Raw Cow’s Milk	
Simazine Concentration (ppb)	%ρ ^1^	Simazine Concentration (ppb)	%ρ ^1^
0.02	18.53	2	20.43
**0.1**	**−18.13**	**10**	**−21.28**
0.2	23.17	20	4.15

^1^ Each mean value was calculate from four runs per day (four replicate per run) repeated for three different days (day-to-day reproducibility). %RSD ≤ 15%; Differences between mean values of Δppm O_2 exp_ and Δppm O_2 blk_ where all statistical significant (*p* < 0.05).

**Table 3:** Means %ρ values from 2 h-bioassays on 10 ppb simazine spiked UHT milk samples.

Milk Fat Content	%ρ ^1^
0	−14.07
1.6	−18.81
3.2	−21.28

^1^ Each mean value was calculate from four runs per day (four replicate per run) repeated for three different days (day-to-day reproducibility). %RSD ≤ 15%; Differences between mean values of Δppm O_2 exp_ and Δppm O_2 blk_ where all statistical significant (*p* < 0.05).

## Supplementary Materials

The following are available online at http://www.mdpi.com/2079-6374/8/4/112/s1, Table S1: Results of chemical analyses of bulk tank raw milk; **Table S2**: Chemical composition of commercial UHT milk; Table S3: Chemical analyses of livestock drinking water; Figure S1: Calibration curve of OD_525nm_ *vs.* cfu/mL; Figure S2: Photograph of the system used for bioassays.
