# Supplementary material for: Towards Simazine Monitoring in Agro-Zootechnical Productions: A Yeast Cell Bioprobe for Real Samples Screening
Source: Biosensors (Basel). 2018 Nov 15;8(4):112. doi: 10.3390/bios8040112 (PMC6316374; doi:10.3390/bios8040112)
Supplement: Supplementary file 1 [file biosensors-08-00112-s001.pdf]

Table S1. Results of chemical analyses of bulk tank raw milk.

Bulk tank raw milk	
Casein (%)[1]	2.51
Fat (%)[1]	3.31
Lactose (%)[1]	4.85
Protein (%)[1]	3.22
Chlorides (mg/mL)[2]	0.680
Urea (mg/dL)[1]	26.7
Inhibiting substances[3]	absent
Aflatoxin M_1 (μg/Kg)[4]	≤ 0.03
pH[5]	6.57
Freezing point (m°C)[1]	-0.523
Total bacteria count (x 1000 CFU/mL)[6]	9
Somatic cell count (x 1000 cells/mL)[7]	235

[1] Test method POS CIP 018 INT rev 11 2015 (Infrared Spectroscopy). [2] Potentiometric titration. [3] AFNOR DSM 28/02 – 02/12 (Delvotest T). [4] Test method POS CHI 038 INT rev 5 2015 (ELISA kit). [5] Test method POS CIP 009 INT rev 7 2015 (Potentiometry). [6] Test method POS CIP 021 INT rev 5 2015 (fluoro-opto-electronic counting) [7] Test method POS CIP 018 INT rev 11 2015 (fluoro-opto-electronic counting).

Table S2. Chemical composition of commercial UHT milk

Composition[1]	Microfiltered full fat UHT milk	Microfiltered semi-skimmed UHT milk	Microfiltered skimmed UHT milk
Total Fat (%w/v)	3.6	1.6	/
Saturated fat (%w/v)	2.5	1.1	/
Carbohydrates (%w/v)	5.0	5.0	5.1
Protein (%w/v)	3.3	3.3	3.4
Sodium (%w/v)	0.10	0.10	0.10

Table S3. Chemical analyses of livestock drinking water.

Livestock drinking water	
pH[1]	7.24
Electrical conductivity @ 20°C (μS/cm)[2]	634
Total dissolved solids (mg/L)[3]	443
Ammonia–nitrogen (mg/L)[4]	<0.03
Iron (μg/L)[5]	<20
Total hardness (°F)[6]	23.4
Nitrites (mg/L)[7]	<0.3
Nitrates (mg/L)[8]	4.1
Sulphates (mg/L)[7]	26
Chlorides (mg/L)[9]	10.4
Fluorides (mg/L)[9]	<0.3
Free active chlorine (mg/L)[10]	<0.03
Manganese (mg/L)[11]	<0.005
Copper (mg/L)[12]	<0.01
Zinc (mg/L)[13]	<0.005
Cadmium (mg/L)[14]	<0.0005
Lead (mg/L)[15]	<0.001
Mercury (mg/L)[16]	<0.0003
Nickel (mg/L)[17]	<0.002
Arsenic (mg/L)[18]	<0.005
Chrome (mg/L)[19]	<0.001
Organophosphate pesticides (mg/L)[20]	<0.01

Organochlorine pesticides (mg/L)[20]	<0.01
Total coliforms (CFU/100 mL)[21]	absent

[1] Test method APAT CNR IRSA 2060 Man 29 2003.[2]Test method APAT CNR IRSA 2030 Man 29 2003. [3]Test method MIC 002/NS rev.1:2009.[4]Test method APAT CNR IRSA 4030 A2 Man 29 2003. [5]Test method APAT CNR IRSA 3160 B Man 29 2003. [6]Test method APAT CNR IRSA 2040 B Man 29 2003. [7]Test method APAT CNR IRSA 4020 Man 29 2003. [8]Test method UNI EN 12014-4:2005. [9] Test method APAT CNR IRSA 4020 Man 29 2003. [10] Test method APAT CNR IRSA 4080 Man 29 2003. [11] Test method APAT CNR IRSA 3010 A Man 29 2003 +APAT CNR IRSA 3190 B Man 29 2003. [12] Test method APAT CNR IRSA 3010 A Man 29 2003 +APAT CNR IRSA 3250 B Man 29 2003. [13] Test method APAT CNR IRSA 3010 A Man 29 2003 + APAT CNR IRSA 3320 A Man 29 2003. [14] Test method APAT CNR IRSA 3010 A Man 29 2003 + APAT CNR IRSA 3120 B Man 29 2003. [15] Test method APAT CNR IRSA 3010 A Man 29 2003 + APAT CNR IRSA 3230 B Man 29 2003. [16] Test method APAT CNR IRSA 3010 A Man 29 2003 + APAT CNR IRSA 3200 A2 Man 29 2003. [17] Test method APAT CNR IRSA 3010 A Man 29 2003 + APAT CNR IRSA 3220 B Man 29 2003. [18] Test method APAT CNR IRSA 3010 A Man 29 2003 + APAT CNR IRSA 3080 A Man 29 2003. [19] Test method APAT CNR IRSA 3010 A Man 29 2003 + APAT CNR IRSA 3150 B1 Man 29 2003. [20] Test method APAT CNR IRSA 5100 Man 29 2003. [21] Test method ISO 9308-1:2017.

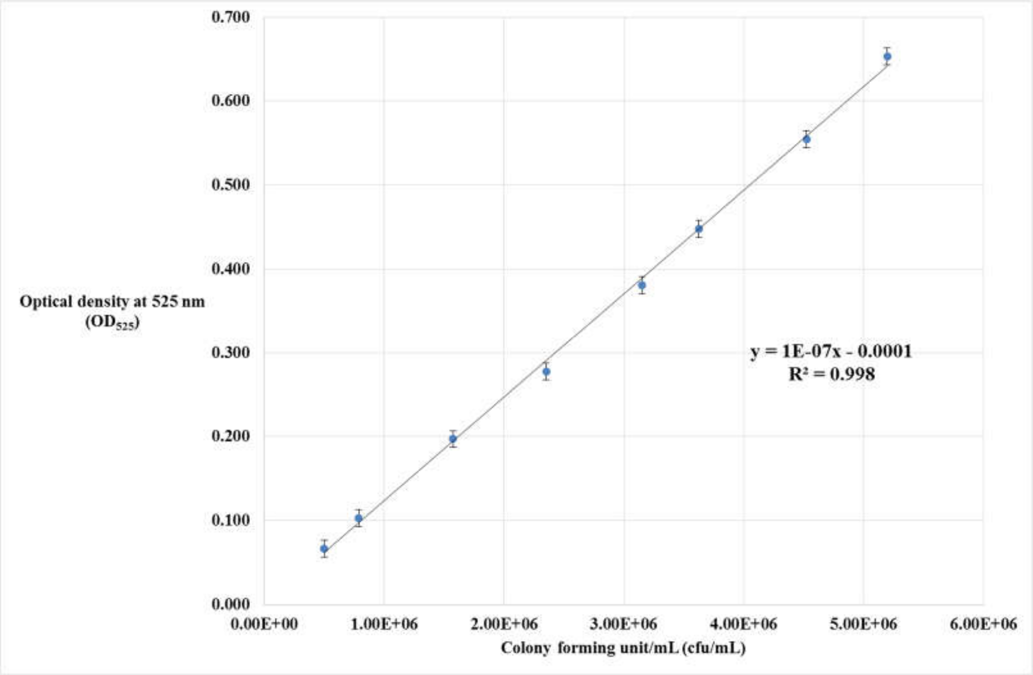

Figure S1. Calibration curve of OD_{525nm} *vs.* cfu/mL. Each point represents a mean value of five replicate tests (%RSD < 5%).

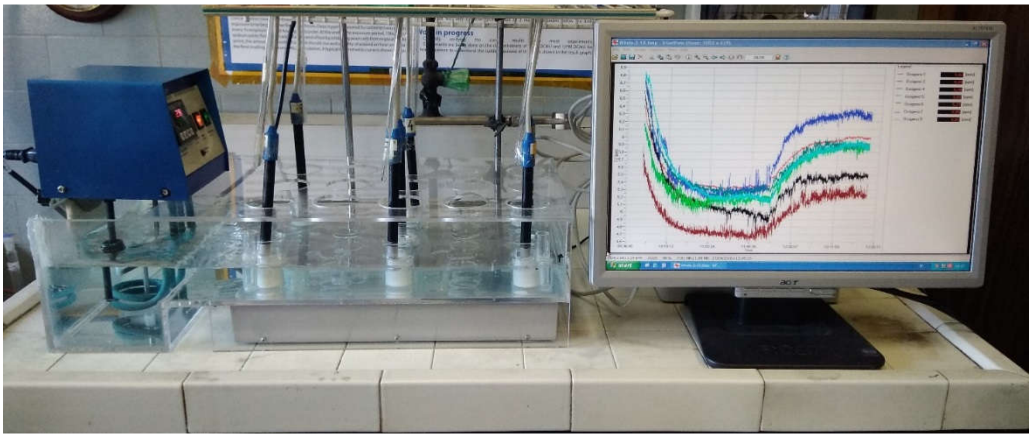

Figure S2. Photograph of the system used for bioassays.
